# How can middle-income countries successfully transition away from international health aid?

**DOI:** 10.1371/journal.pmed.1004794

**Published:** 2025-11-06

**Authors:** Osondu Ogbuoji, Ipchita Bharali, Justice Nonvignon, Gavin Yamey

**Affiliations:** 1 Center for Policy Impact in Global Health, Duke Global Health Institute, Duke University, Durham, North Carolina, United States of America; 2 Duke Margolis Institute for Healthcare Policy, Duke University, Durham, North Carolina, United States of America; 3 Department of Population Health, Duke University School of Medicine, Duke University, Durham, North Carolina, United States of America; 4 Management Sciences for Health, Arlington, Virginia, United States of America; 5 School of Public Health, University of Ghana, Lagon, Ghana; 6 Sanford School of Public Policy, Duke University, Durham, North Carolina, United States of America

## Abstract

Pertinent to recent international health aid cuts, this Perspective discusses how middle-income countries can prepare and succeed in transitioning away from external aid, highlighting three factors important factors: Effective leadership, using domestic resources to close the funding gap, and realigning country systems to new sources of domestic funding.

## Teaser

Recent research has examined factors contributing to the successful transition of middle-income countries away from international health aid. Three factors are especially important: effective leadership, using domestic resources to close the financing gap created by loss of aid, and realigning country systems to new sources of domestic funding.

## Introduction

Recent cuts to global health aid have reignited the debate about the prospects of low- and middle-income countries (LMICs) successfully transitioning away from international health aid to domestic resources for health. From January to June 2025, the United States (US), the United Kingdom (UK), and other high-income countries made abrupt and drastic cuts to their health aid budgets that threaten progress in public health in recipient countries if not managed carefully. Countries also risk losing access to concessional aid when they experience economic growth and transition from low-income country (LIC) status to middle-income country status. In general, aid-receiving countries can experience both planned and abrupt aid cuts, which can be devastating.

In a previous article, we argued that this transition out of health aid, along with other transitions faced by LMICs, can negatively affect population health if not properly managed [1]. These effects include increasing health inequalities arising from worsening health outcomes in marginalized populations and pockets of poverty within these countries [1,2]. Since then, extensive research on transitions has been conducted, examining evidence on the factors that contribute to successful transitions, where funding gaps are bridged, program interruptions are minimized, and gains in population health outcomes are preserved [3]. Among the many factors that contribute to successful transitions, three stand out: (i) Effective leadership; (ii) using domestic resources to close the financing gap created by the transition; (iii) realignment of country systems to new sources of domestic funding.

## Effective leadership

Effective leadership is essential for a successful transition. For leadership to be effective, it must be jointly led by both the recipient and the donor, as unilateral decision-making rarely works. It should also be transparent, with mutually agreed milestones and clear communication about progress and challenges. Leadership is important because transition is not a linear process; it often requires revisiting and adjusting plans. Effective leadership secures political commitment, legislative and policy support for transition, and domestic ownership of health programs.

One common tool to promote joint, transparent, and collaborative leadership is a transition plan. The best transition plans are developed collaboratively by all stakeholders, include a transition readiness assessment, have clear milestones, have buy-in from the highest levels of political leadership, and address funding gaps and system reforms [3,4]. Examples of successful transitions that demonstrated strong leadership include: The transfer of the Avahan HIV program from the Gates Foundation to the Government of India, where both parties worked collaboratively to develop, adjust, and monitor transition plans [5]; the Government of Swaziland collaborating with several partners, including the Clinton Health Access Initiative, to develop a price benchmarking tool to support long-term procurement of antiretroviral medicines post-transition [6]; and sustained improvements in HIV prevention efforts following the exit of the Global Fund to Fight AIDS, Tuberculosis and Malaria (the Global Fund) from Estonia, where there was proactive development and adoption of a detailed transition plan by a national multistakeholder group [7].

However, abrupt transitions—such as those precipitated by the recent aid cuts by the US and the UK—can derail any potential for joint leadership, highlighting the importance of recipient countries being deliberate about planning for transitions to circumvent negative impacts. When countries have functioning coordination platforms where different donors rally behind the country’s leadership, this provides an opportunity to drive the transition planning agenda proactively, with the engagement of all donors. In that case, the abrupt withdrawal of one or a few donors would not likely derail the joint transition planning process.

## Closing the financing gap created by transitions

Countries have approached closing financial gaps created by transitions in different ways. The three most common include (i) identifying new sources of domestic public financing, (ii) reallocating existing resources through budget adjustments, and (iii) identifying alternative private sector sources.

For example, even as Kenya started preparing to transition away from health aid, the health sector showed high levels of donor dependency. A handful of donors, like the US, the UK, the Global Fund, and Gavi, the Vaccine Alliance (Gavi), contributed more than 90% of all health aid, supporting major health programs in the country. In 2020−2021, the US President’s Emergency Plan for AIDS Relief (PEPFAR) financed nearly 60% of all of HIV programs in Kenya [8]. This made Kenya highly vulnerable to donor transitions and exits. To raise new funds, Kenya introduced an airline tax and allocated 1% to HIV/AIDS programming. Additionally, the donors and country leadership worked together to develop elaborate transition plans to shift towards domestic health financing sources. These include the Kenya AIDS Strategic Framework II 2020/21–2024/25 and Kenya’s Operational Plan for Enhancing Country Readiness to Sustain a Resilient HIV Response Beyond 2030. Kenya has also increased budgetary allocation to health (an 8.74% increased allocation in 2025−2026 compared to the previous fiscal year) and introduced mandatory social health insurance to generate more domestic revenues for health [9].

Prior to its exit from funding family planning programs in Latin America, USAID worked collaboratively with host countries to ensure that there were budgetary line items for contraceptives and other necessary program materials. Efforts to ensure a structured transition included the use of predefined financial and technical benchmarks, phased-out support starting with contraceptive supplies followed by phased technical assistance, and building self-sufficiency through political and financial commitment [10].

Similarly, in Albania, Gavi and the government worked to ensure a separate line item in the national budget for vaccines. These efforts were complemented by institutionalization of the Gavi-supported budget planning process to improve budgeting and procurement processes and setting up post-transition coordination mechanisms for continued collaboration [11].

To offset the loss of funds after USAID’s withdrawal from family planning programs in Ecuador, civil society organizations (CSOs) were trained in revenue generation methods. This training led to income-generating activities such as providing pediatric care and dental services, supporting financial sustainability [12].

## System realignment to new sources of funding

The aforementioned successful initiatives to close funding gaps during and after transitions were driven by strong governance, commitment to financial sustainability, and institutionalized efforts to improve budgeting and prioritization of health. Realignment of country systems to fit the new funding ecosystem is a crucial but often overlooked factor that contributes to successful transitions. This realignment often aims to achieve full or partial integration of programs previously funded by donors into the host country’s health financing and health service delivery systems. Failure to do this could lead to program interruptions even when funding gaps are closed. System realignment can take different forms; in Box 1, we highlight three common forms.

**Box 1. Three common forms of system realignment as a result of donor transition**

1. **Establishing new policies to enable contracting and financial flows**: Before the exit of the Global Fund from HIV prevention programs targeting key populations in Macedonia, it collaborated closely with the Ministry of Health to develop a system that would allow the government to engage with CSOs. Without this system, it would have been impossible for the government to contract CSOs to continue providing HIV prevention services [13].
2. **Strengthening the capacity of existing institutions to manage funds and programs:** In El Salvador, the Global Fund, the United Nations Development Programme, and the government worked to enhance the government’s ability to manage program funds and conduct program management [14]. Similarly, in Bangladesh, the government and the Global Fund collaborated to strengthen the government’s ability to manage an effective supply chain system, thereby reducing antiretroviral stockouts in the Modhumita HIV prevention and treatment program.
3. **Reforms to enable effective use of existing highly skilled human resources:** These reforms could involve integrating experts previously funded by donors into government or other local institutions. The challenge is often that donor salaries and benefits surpass public sector offerings, making retention difficult. In Botswana, PEPFAR once funded 150 planning and strategic information officers to support the country’s HIV response. However, when PEPFAR shifted towards greater country ownership, staff turnover increased, resulting in significant gaps in technical capacity.

Although the three factors discussed in our piece are not the only ones affecting transition success, they are nonetheless important and have a disproportionately large impact on the likelihood of success. Successful transition experiences demonstrate that leadership, planning, and pre-transition investments in a country’s financial, technical, logistical, and service delivery capacities are crucial to ensuring a smooth transition. Addressing institutional gaps is just as important as addressing funding gaps.

Our research also revealed cases where transitions are almost always unsuccessful. The two most common are countries facing severe political instability and LICs that cannot bridge funding gaps. Political instability, such as that seen in fragile and conflict-affected states, appears to hinder successful transition. This may be due to the inability of such countries to inspire the necessary leadership for transition or because their leaders prioritize other issues.

Similarly, LICs are less likely to succeed in donor transitions than middle-income countries because (i) they significantly underspend on health; and (ii) their health sectors have high levels of donor dependency; 20 of the 25 LICs listed in the World Health Organization’s Global Health Expenditure Database depend on donors for 20% or more of their total healthcare spending. LICs, therefore, lack the resources to close the funding gap needed for a successful transition.

## Conclusions

Lessons from previous donor transitions show that factors like governance, economic resilience, and financing systems influence a country’s preparedness to transition successfully. Understanding these dynamics and developing tailored transition strategies can mitigate risks of backsliding in health outcomes.

Although donor exits can be catastrophic, this is not inevitable. Country examples have shown that adopting strong leadership and coordination principles, careful attention to closing funding gaps created by donor exits, and proper realignment of country systems to reflect the new funding realities can significantly reduce the negative impacts of donor exits.

## Abbreviations

CSOs: civil society organizations
LIC: low-income country
LMICs: low- and middle-income countries
PEPFAR: US President’s Emergency Plan for AIDS Relief
