# Correction: How can middle-income countries successfully transition away from international health aid?

**DOI:** 10.1371/journal.pmed.1005018

**Published:** 2026-03-23

**Authors:** Osondu Ogbuoji, Ipchita Bharali, Justice Nonvignon, Gavin Yamey

In the Closing the financing gap created by transitions subsection of the Effective leadership, the fifth sentence of the second paragraph should not have been included in the article. The correct paragraph is: For example, even as Kenya started preparing to transition away from health aid, the health sector showed high levels of donor dependency. A handful of donors, like the US, the UK, the Global Fund, and Gavi, the Vaccine Alliance (Gavi), contributed more than 90% of all health aid, supporting major health programs in the country. In 2020−2021, the US President’s Emergency Plan for AIDS Relief (PEPFAR) financed nearly 60% of all of HIV programs in Kenya [8]. This made Kenya highly vulnerable to donor transitions and exits. Additionally, the donors and country leadership worked together to develop elaborate transition plans to shift towards domestic health financing sources. These include the Kenya AIDS Strategic Framework II 2020/21–2024/25 and Kenya’s Operational Plan for Enhancing Country Readiness to Sustain a Resilient HIV Response Beyond 2030. Kenya has also increased budgetary allocation to health (an 8.74% increased allocation in 2025−2026 compared to the previous fiscal year) and introduced mandatory social health insurance to generate more domestic revenues for health [9].
